# Isolation of bifidobacteria for blood group secretor status targeted personalised nutrition

**DOI:** 10.3402/mehd.v23i0.18578

**Published:** 2012-06-18

**Authors:** Harri Mäkivuokko, Pirjo Wacklin, Marjorie E. Koenen, Karoliina Laamanen, Noora Alakulppi, Koen Venema, Jaana Mättö

**Affiliations:** 1 Finnish Red Cross Blood Service, Helsinki, Finland; 2 TNO, Zeist, The Netherlands

**Keywords:** gastrointestinal simulation, probiotic screening, Bifidobacterium, intestinal, ABO blood group, secretor, non-secretor

## Abstract

**Background:**

Currently, there is a constant need to find microbial products for maintaining or even improving host microbiota balance that could be targeted to a selected consumer group. Blood group secretor status, determining the ABO status, could be used to stratify the consumer group.

**Objective:**

We have applied a validated upper intestinal tract model (TIM-1) and culturing methods to screen potential probiotic bacteria from faeces of blood secretor and non-secretor individuals.

**Design:**

Faecal samples from healthy volunteers were pooled to age- and sex-matched secretor and non-secretor pools. Faecal pools were run through separate TIM-1 simulations, and bacteria were cultivated from samples taken at different stages of simulations for characterisation.

**Results:**

Microbes in secretor pool survived the transit through TIM-1 system better than microbes of non-secretor pool, especially bifidobacteria and anaerobes were highly affected. The differences in numbers of bifidobacteria and lactobacilli isolates after plate cultivations and further the number of distinct RAPD-genotypes was clearly lower in non-secretor pool than in secretor pool.

**Conclusions:**

In the present study, we showed that microbiota of secretor and non-secretor individuals tolerate gastrointestinal conditions differently and that a combination of gastrointestinal simulations and cultivation methods proved to be a promising tool for isolating potentially probiotic bacteria.

The human intestinal tract is colonised with several hundred bacterial species, whose total number can exceed trillions of microbial cells in the colon. The microbiota has an important role in human health. It contributes to the maturation of the gut tissue, host nutrition, pathogen resistance, epithelial cell proliferation, host energy metabolism, and immune response (1, 2). That host genes might play a role in determining the composition of the gut microbiota has been supported by twin studies (3) and a few pioneer studies on specific genes, e.g. *fucosyltransferase*-2 (*FUT2*) (4). Some pathogenic intestinal microbes, e.g. *Helicobacter pylori* and several other species of bacteria and viruses, have shown to use ABO blood group antigens as adhesion receptors (5). Some microbes, e.g. bifidobacteria and *Bacteroides thetaiotaomicron*, are also able to utilise blood group antigens or glycans found in ABO and Lewis antigens (6, 7).

The ABO blood group antigens are not present in the mucus of all individuals. These individuals, said to have the ‘non-secretor’ blood group, do not have the functional *FUT2* gene needed in the synthesis of secreted blood group antigens (8). Hence, they do not have ABO antigens in their secretions and mucosa, while those with blood group ‘secretor’ have the antigens. In most populations, the frequency of non-secretor individuals is substantially lower than that of secretor status; about 15–26% of Scandinavians are non-secretors (9). The secretor/non-secretor status can be regarded as a normal blood group system and the phenotype can be determined using standard blood banking protocols (8). The genotype that is causing the non-secretor (NSS) phenotype in European populations has been identified as a major mutation in the *FUT2* gene (10). The non-secretor phenotype has been demonstrated to be genetically associated with Crohn's disease (11), resistance to Norovirus infection (12), experimental vaginal candidiasis (13), increased risk for asthma (14), urinary tract infections (15), and animal haemorrhagic disease virus (16).

The beneficial effects of certain microbial species/strains on maintaining or even improving of gut balance and the growing evidence of their health effects on intestinal inflammatory diseases have caused a great interest in modulation of gut microbiota, and recently also on modulation of oral, vaginal or skin microbiota. Gut microbiota can be modulated by taking probiotics belonging mainly to *Bifidobacterium* and *Lactobacillus* genera. Many probiotic supplements and products currently on the market are ineffective in promoting the desired health effects among most individuals. Thus, there is a continuous need for microbial and/or probiotic products that are able to mediate the health effects of the microbes more efficiently. An important criterion for the selection of probiotic bacteria to be used in food products or formulations in relation to health promotion is the survival and activity of these microorganisms in the gastrointestinal (GI) tract of the consumer. In order to investigate the survival of probiotic bacteria in the stomach and small intestine in humans, intubation methods are available. However, these *in vivo* methods are laborious, expensive, and meet ethical constraints. Therefore, validated *in vitro* methods should be performed, at least for the first selection of strains. In our study, we investigated the difference in the GI survival of intestinal microbiota obtained from blood group secretor and non-secretor individuals by applying the TIM-1 system (TNO intestinal model-1) (17) followed by selective culturing as an approach for microbiota characterisation. This approach enables the isolation of secretor/non-secretor-specific potentially probiotic bacteria.

## Present investigation

### Sample collection

Faecal and blood samples were collected from a group of healthy volunteers; blood samples were processed directly, and faecal samples were processed under anaerobic atmosphere. Of the blood samples, the presence or absence of the mutation responsible for non-secretor genotype was determined by using sequencing or a qPCR method as described in Refs. (10, 18) at Haartman Institute Sequencing core facility and at the Technology Centre, Institute for Molecular Medicine Finland (FIMM), University of Helsinki, Helsinki. Based on this SNP-data, a secretor- and a non-secretor-pool were formed amongst the volunteers by selecting age- and sex-matched pairs from the respective groups, resulting in a total of 11 in both groups. Frozen faecal samples were pooled based on the grouping to form a secretor (S) and a non-secretor (N) faecal pool.

### Isolation of new strains by TIM-1 model

The TIM-1 (TNO *in vitro* GI model-1) of the stomach and small intestine (17) simulates to a high degree the successive dynamic processes in the stomach and the small intestine allowing sampling from several sites of the simulated GI tract in time. The model offers the possibility to simulate very closely the pH curves and the concentrations of enzymes in the stomach and small intestine, the concentrations of bile salts in the different parts of the gut, and the kinetics of passage of food through the stomach and intestine. It has been validated for the survival of probiotic microorganisms (17) and shows such a high *in vitro*–*in vivo* correlation that the system can be used for predicting survival of bacteria during transit through the GI tract.

In this study, the TIM-1 model was used with faecal samples to facilitate the isolation of microbes with good survival under GI tract conditions. The screening of the target bacteria was performed by adding the faecal slurries to TIM-1 and plate culturing the ileum effluent to grow the surviving bacteria. We collected isolates from bifidobacteria, lactobacilli, bacteroides, and general anaerobes and focused on characterising isolated bifidobacteria strains to deeper detail.

The frozen faecal samples were taken from the freezer and directly transferred into the anaerobic cabinet, where they were mixed together with artificial saliva and water. The non-secretor pool had in total 12.1 g and the secretor pool in total 9.8 g faecal material. A mixture of 95 ml artificial saliva and 200 ml water was prepared, and in this mixture, the total amount of the faecal samples of one pool was mixed. A 1 ml sample was taken from each mixture for plating. The bacterial slurries were then introduced into the gastric compartment containing 5 ml gastric residue.

The experiments in the TIM-1 model were performed under the average physiological conditions as found in the healthy human GI tract (19). The gastric emptying, intestinal residence time, and gastric and duodenal pH-curve mimicked the situation as found in humans in a fasted state. The concentrations of electrolytes, enzymes, bile, bile salts, and pancreatic juice were adjusted at the average concentrations as described for healthy humans.

During the TIM-1 run, ileum effluent was sampled. The simulated ‘ileocaecal valve’ delivered the intestinal contents into a sampling bottle with 250 ml of diluent (cystein tryptone salt solution, containing 1.0 g tryptone, 8.50 g NaCl, and 0.30 g L-cystein hydrochloride per litre) at 10 (±2)°C. In this way, the sample was diluted to minimise the effect of bile and pH after the passage through the TIM-1 system on the remaining surviving bacteria. Every 60 min, the collected volume was measured and sampled. A 15-ml aliquot of the ileal effluents (Ie) of time points Ie-1 (60–120 min), Ie-2 (120–240 min), and Ie-3 (240–300 min) was collected for direct plating. Thus, in each TIM-1 experiments, four samples (intake, Ie-1, Ie-2, and Ie-3) were plated. The growth media and conditions used for cultivation of each species are presented in Table 1. Representative colonies were collected from culture plates for further analysis.

**Table 1:** Growth media and conditions for bacteria cultivation

Agar medium	For which bacteria	Incubation temp. (°C)	Incubation aerobic/anaerobic	Incubation time (h)
Beerens	Bifidobacteria	37	Anaerobic	72
RB	Bifidobacteria	37	Anaerobic	72
Rogosa	Lactobacilli	37	48 h aerobic/24 h anaerobic	72
LAMVAB	Lactobacilli	37	Anaerobic	72
RCBA	Anaerobes	37	Anaerobic	72
BBE	Bacteroides	37	Anaerobic	72

RB (raffinose-bifìdobacterium agar), LAMVAB (*Lactobacillus* anaerobic MRS with vancomycin and bromocresol), RCBA (reinforced Clostridial agar with China blue and horse blood) and BBE (bacteroides bile esculin agar).

### Characterisation of isolates and strain properties

Isolates were collected from each of the targeted culture plate types and stored in skimmed milk for further characterisation. Bacteroides and anaerobes were not analysed further in this study (Table 3). The bifidobacteria and lactobacilli isolates were first analysed using random amplification of polymorphic DNA (RAPD) to rapidly and roughly screen the different strains amongst the isolates. DGGE-analysis was performed with the RAPD-identified bifidobacteria and lactobacilli strains as the primary species identification method (Table 3). At this point, we focused solely on bifidobacteria and continued their identification by 16S sequencing of bacterial DNA. Not all 16S sequence measurements were successful, and ID based on DGGE-phenotyping was used in these cases (Table 3).

The DGGE-analysis was performed as described previously in Ref. (4). Briefly, the DNA from bacterial cultures was collected in log-phase and DNA was extracted by using the FASTDNA^®^ SPIN KIT FOR SOIL (Qbiogene, USA). The partial 16S rRNA gene was PCR amplified with *Bifidobacterium* specific primers, run in DGGE gel with denaturing gradient from, stained with SYBRSafe, and documented with an UV-table and AlphaImager HP (Kodak, USA) imaging system.

RAPD was performed as described in Ref. (20). The isolates were lysed followed by PCR amplification with a random primer set OPA-2 (bifidobacteria) or OPA-3 (lactobacilli). The PCR products were separated by gel electrophoresis and the fingerprints of the isolates from the same individual were compared visually.

Substrate utilisation and/or enzyme activation were determined with two techniques: OD-measurement with Bioscreen (Growth Curves Ltd., Finland) and colour reaction with Rosco Diatabs (Rosco Diagnostica A/S, Denmark). Prior to both measurements, the bifidobacteria strains were grown for 48 h under anaerobic conditions in +37°C first on RCM-agar-plates and subsequently in RCM-broth. In Bioscreen measurements after precultivation, a 5% inoculum of each strain was cultivated in duplicate in 0.5% fucose-, lacto-n-biose-, or glucose-solutions mixed in low carbohydrate general edible medium (21) under anaerobic conditions at +37°C with slow shaking for 48 h. Starting- and end-point OD-measurements were performed with Bioscreen. The average growth of 0.5% glucose was deducted from 0.5% fucose and lacto-n-biose results. In Rosco Diatabs, the manufacturer's instructions were followed for both substrate utilisation and enzyme activation disks. The precultivated bacteria were washed with PBS and diluted to OD 1.0 in PBS, 100 µl of each dilution was pipetted to 96-well plate, and respective Rosco Diatabs disks were placed to the wells. The plate was cultivated in +37°C under anaerobic condition for 24 h, and the results were determined by visual inspection of the colour reaction. Rosco Diatabs Oxgall disks were used to test the bile acid tolerance of the screened bifidobacteria strains. Tolerance for bile acid was tested using Rosco Diatabs Oxgall as instructed by the manufacturer by plating PBS-diluted bifidobacteria colonies on RCM-agar together with Oxgall disks. The tolerance was determined by measuring the inhibition zones surrounding the disks. Zones with maximum diameter of 9 mm were determined as resistant (R) and rest as sensitive (S).

## Results

A difference in the survival of faecal bacteria (especially bifidobacteria and total anaerobes) in the TIM-1 model was observed between the non-secretor and secretor sample pools (Table 2), although the numbers of bacteria in the intakes were nearly identical. From the non-secretor pool, fewer bacteria survived the transit through the TIM-1 system than from the secretor pool, although the lactobacilli count (using Rogosa-agar) was the only count higher in the non-secretor pool. The highest differences in survival, up to more than 10 times higher cfu's, were detected in secretor pool bifidobacteria and anaerobes. Also, the visual inspection of the culture plates suggested a difference in the diversity and microbiota composition between the two sample pools. The difference in the number of obtainable bifidobacteria and lactobacillus isolates and the number of distinct genotypes (determined by RAPD) was clearly lower in the non-secretor pool than in secretor pool (Table 3), further supporting the difference in the intestinal survival properties of the microbiota of secretor and non-secretor individuals.

**Table 2:** Survival of intestinal bacteria (number of cfu's) in the TIM-1 samples in non-secretor pool and secretor pool

Non-secretor	Total number of cfu in the samples						Secretor	Total number of cfu in the samples					
			
Sample	Beerens	RB	Rogosa	LAMVAB	RCBA	BBE	Sample	Beerens	RB	Rogosa	LAMVAB	RCBA	BBE
intake	4.1E + 08	3.2E+09	3.0E+09	4.1E+07	2.9E+10	2.2E+09	intake	6.5E+08	3.4E+09	8.4E+09	1.3E+07	2.3E+10	3.4E+08
Ie-1	1.7E+05	1.9E+07	1.9E+07	3.4E+03	4.8E+07	0.0E+00	Ie-1	1.0E+07	4.8E+07	4.1E+07	3.5E+04	4.1E+08	0.0E+00
Ie-2	2.4E+05	2.1E+07	1.3E+07	6.8E+03	1.6E+08	0.0E+00	Ie-2	5.1E+06	5.1E+07	3.3E+07	1.7E+04	1.3E+09	3.4E+06
Ie-3	1.4E+05	2.4E+07	1.6E+07	3.5E+03	5.2E+08	0.0E+00	Ie-3	5.4E+06	6.5E+07	3.0E+07	2.4E+04	4.1E+09	0.0E+00
Total survival	5.5E+05	6.5E+07	4.8E+07	1.4E+04	7.3E+08	0.0E+00	Total survival	2.1E+07	1.6E+08	1.0E+08	7.5E+04	5.8E+09	3.4E+06
% Survival	0.1	2.0	1.6	0.0	2.5	0.0	% Survival	3.2	4.8	1.2	0.6	25.0	1.0

**Table 3:** Bifidobacteria and lactobacilli isolates collected from the TIM-1 model experimentation sample plates

	No. of RAPD types (no. of isolates)		Species	
		
Group	Secretor	Non-secretor	Secretor	Non-secretor
Bifidobacteria	31 (276)	11 (196)	6	5
Lactobacilli	21 (126)	8 (65)	9	6

In growth experiments with specific substrates (lacto-n-biose and fucose), eight of the 36 bifidobacterial strains utilised fucose and six strains used lacto-n-biose slightly better than glucose (Table 4). The S-pool users belong to both *B. adolescentis* and *B. catenulatum* species and the N-pool to *B. adolescentis* species. In the Rosco enzyme activity and substrate utilisation tests, *B. animalis* species were detected to be the best users of the studied mono/disaccharides, and along with *B. adolescentis* and *B. catenulatum* species having the broadest enzyme arsenal at their disposal. *B. longum* species, followed by the *B. adolescentis* species had the lowest utilisation capabilities of the studied mono/disaccharides, and several *B. longum* species lacked the β-galactosidase activity. All in all, N-pool strains were detected to be more enzymatically active compared to S-pool strains. α-Fucosidase activity was not found in any of tested strains.

**Table 4:** Substrate utilization, enzyme activity, and bile tolerance of bifidobacteria isolated from secretor and non-secretor individuals

	No. of positive isolates/total no. of isolates (%)											
	
	Growth*		Rosco Diatabs									
		
Species	Fucose	Lacto-n-biose	Glucose	Lactose	Sucrose	Mannose	α-Glucosidase	β-Glucosidase	α-Galactosidase	β-Galactosidase	α-Fucosidase**	Bile resistance
Secretor												
*Bifidobacterium* sp.	0/1	0/1	1/1	1/1	1/1	1/1	1/1	1/1	1/1	1/1	–	0/1
*B. adolescentis*	4/8	2/8	6/8	8/8	7/8	3/8	8/8	8/8	8/8	8/8	0/3	2/8
*B. catenulatum*	2/4	2/4	4/4	4/4	4/4	3/4	4/4	3/4	4/4	4/4	0/1	2/4
*B. longum*	0/8	0/8	6/8	8/8	4/8	5/8	8/8	3/8	8/8	6/8	0/3	0/8
*B. animalis*	0/4	0/4	4/4	4/4	4/4	4/4	4/4	3/4	4/4	4/4	0/1	4/4
All	6/25 (24%)	4/25 (16%)	21/25 (84%)	25/25 (100%)	20/25 (80%)	16/25 (64%)	25/25 (100%)	18/25 (72%)	25/25 (100%)	23/25 (92%)	0/8 (0%)	8/25 (32%)
Non-secretor												
*Bifidobacterium* sp.	0/1	0/1	0/1	1/1	0/1	1/1	1/1	1/1	1/1	1/1	–	1/1
*B. adolescentis*	2/3	2/3	2/3	3/3	3/3	3/3	3/3	3/3	3/3	3/3	0/3	1/3
*B. catenulatum*	0/2	0/2	2/2	2/2	2/2	1/2	2/2	2/2	2/2	2/2	–	2/2
*B. longum*	0/4	0/4	3/4	4/4	2/4	2/4	4/4	4/4	4/4	4/4	0/3	1/4
*B. animalis*	0/1	0/1	1/1	1/1	1/1	1/1	1/1	1/1	1/1	1/1	0/1	1/1
All	2/11 (18%)	2/11 (18%)	8/11 (73%)	11/11 (100%)	8/11 (73%)	8/11 (73%)	11/11 (100%)	11/11 (100%)	11/11 (100%)	11/11 (100%)	0/7 (0%)	6/11 (55%)

* Bioscreen growth marked positive if (OD after growth with 0.5% substrate–OD after growth with 0.5% glucose) > 0.

** Due product shortage, α-fucosidase production was measured only from total of 15 strains.

– States for not measurement.

Five *B. animalis*, three *B. adolescentis*, two *B. catenulatum*, and one *B. longum* strains were found to tolerate bile and 55% of the N-pool strains tolerated bile well compared to 32% of the S-pool.

## Conclusions

In the present study, we show the difference in the GI survival properties of the faecal microbiota of blood group secretor and non-secretor individuals. In addition, we present a rapid approach for screening potential personalised probiotic strains from human faecal samples. We combined the traditional plate cultivation, still the most efficient way to collect live bacterial isolates, with an accurately upper GI tract conditions simulating model.

The faecal samples used in this study were collected from healthy individuals and pooled according to donor's blood group secretor or non-secretor status. The secretor individuals express by definition ABO blood group antigens in, e.g. their GI secretions, whereas non-secretors express only Lewis a-antigen. Our group recently published a novel finding stating non-secretors and secretors have marked differences in their faecal bifidobacteria populations, non-secretors having both clearly lower overall counts, and fewer bifidobacteria species present (4). In the current study, we were able to show that the secretor status associated difference in the microbial species composition reflects also the phenotypic properties of the intestinal bacteria especially those influencing the tolerance to upper GI conditions (22).

We utilised our previous findings as a basis to screen secretor status-specific probiotic strains with a novel approach. The TIM-1 system has been widely applied to study the upper GI survival properties of single bacterial strains (17, 19). In our approach, we utilised the TIM-1 for the preselection of potentially acid- and bile-tolerant intestinal bacteria. The model offers the possibility to study the survival and behaviour of probiotic and pathogenic microorganisms in the stomach and small intestine, and the results can be used to predict the survival of probiotic bacteria in the human intestinal tract. The isolate cultivation results supported our hypothesis well, as the bacteria in the non-secretor faecal pool had significantly lower survival rate in the TIM-1 simulation compared to the secretor pool bacteria. Especially non-secretor bifidobacteria and anaerobes survived very poorly compared to respective species in secretor pool. However, only *B. animalis* species were isolated in the ileal effluents in both simulations, thus being only strains able to withstand the harsh conditions of the upper GI tract. Due to the excellent acid and bile tolerance, *B. animalis* is widely used in probiotic products in Europe.

Interestingly, the users of lacto-n-biose were found only amongst *B. adolescentis* (two in both S- and N-pools) and *B. catenulatum* (two in S-pool). As lacto-n-biose is the basic backbone of all ABO blood group glycan structures, these strains, and especially two strains tolerating bile (N-pool *B. adolescentis* and S-pool *B. catenulatum*), might be potential probiotic *Bifidobacterium* candidates aimed to utilise ABO blood group structures and warranting further studies.

In conclusion, we showed that the tolerance of the intestinal microbiota to upper GI conditions differs between secretor and non-secretor individuals, secretor bifidobacteria surviving in larger number, whereas non-secretor bifidobacteria had broader glycolytic activity and better bile tolerance. In addition, we compiled a selective pretreatment and culturing approach, which appears to be a promising tool for isolation of potentially probiotic bacteria.
